# Age-related histone H3.3 accumulation associates with a repressive chromatin in mouse tibialis anterior muscle

**DOI:** 10.1186/s12576-024-00935-2

**Published:** 2024-09-14

**Authors:** Ryo Masuzawa, Hemilce Karina Rosa Flete, Junya Shimizu, Fuminori Kawano

**Affiliations:** Graduate School of Health Science, Matsumoto University, 2095-1 Niimura, Matsumoto, Nagano 390-1295 Japan

**Keywords:** Aging, Epigenetics, Histone modification, Chromatin immunoprecipitation, Exercise

## Abstract

The present study aimed to investigate age-related changes in histone variant H3.3 and its role in the aging process of mouse tibialis anterior muscle. H3.3 level significantly increased with age and correlated with H3K27me3 level. Acute exercise successfully upregulated the target gene expression in 8-wk-old mice, whereas no upregulation was noted in 53-wk-old mice. H3K27me3 level was increased at these loci in response to acute exercise in 8-wk-old mice. However, in 53-wk-old mice, H3.3 and H3K27me3 levels were increased at rest and were not affected by acute exercise. Furthermore, forced H3.3 expression in the skeletal muscle of 8-wk-old mice led to a gradual improvement in motor function. The results suggest that age-related H3.3 accumulation induces the formation of repressive chromatin in the mouse tibialis anterior muscle. However, H3.3 accumulation also appears to play a positive role in enhancing skeletal muscle function.

**Supplementary Information:**

The online version contains supplementary material available at 10.1186/s12576-024-00935-2.

## Background

Sarcopenia is an age-related pathogenic condition that is characterized by the progressive loss of skeletal muscle mass and function. Both intrinsic factors, such as abnormal secretion, motor neuron loss, mitochondrial dysfunction, and insulin resistance, as well as and extrinsic factors, such as inactivity and low nutrition, underlie the severe loss of skeletal muscle mass in aged individuals [1]. While these physiological changes are hallmarks of the late phase of skeletal muscle senescence, a substantial loss of skeletal muscle mass is noted at earlier life stages. Previous studies [2, 3] reported that the decline in skeletal muscle mass in men started as early as 40 years. On the other hand, skeletal muscle mass was more stable in women, and decreased only slightly among women in the higher percentiles. Skeletal muscle aging is a long process and takes a considerable duration of one’s life span to disrupt the well-coordinated cellular homeostasis. Hence, skeletal muscle senescence is thought to have a long “pre-symptomatic state”. It has been hypothesized that the biological framework that triggers the disruption of cellular functions is coordinated by both genetic and post-maturity environmental factors during middle age. However, the precise mechanisms underlying the progression of the “pre-symptomatic state” in skeletal muscle with age are poorly understood.

Epigenetic alterations, namely DNA methylation and histone modifications, are associated with age-related disorders of the skeletal muscle. Zykovich et al. [4] reported that DNA hypermethylation is associated with aging in human skeletal muscle and is pronounced at loci related to myotube fusion, oxidative phosphorylation, and voltage-gated calcium channels. Consistently, Voisin et al. [5] reported differentially methylated CpG sites in the skeletal muscles of younger and older subjects. However, the methylation levels of these CpG sites in muscles from older subjects approached the methylation levels of those from younger subjects following exercise training. Furthermore, Antoun et al. [6] reported that sarcopenia-associated differentially methylated CpG sites are enriched in regions with overlapping sites for H3K27me3 and EZH2 binding motifs. Additionally, treatment with GSK343, an EZH2 inhibitor, reportedly improved mitochondrial respiration in human primary myoblasts, and was associated with altered DNA methylation at related loci. Although these studies indicate a close relationship between DNA methylation and aging of skeletal muscles, little is known with respect to changes in histone distribution and modifications in skeletal muscles during aging.

Nucleosomes are composed of 147 base pairs of DNA that are wrapped around a histone octamer comprised of two copies of the following core histone protein: H2A, H2B, H3, and H4. Histone H3.3 is a non-canonical variant of histone H3, and has been reported to play a key role in myogenic differentiation. Harada et al. [7] demonstrated that myogenic regulatory factor MyoD-dependent incorporation of H3.3 into skeletal muscle genes in fibroblasts increased the distribution of histone H3 that was bivalently modified by H3K4me3 and H3K27me3. Additionally, they reported bivalent modifications on H3.3-incorporated skeletal muscle genes prior to embryonic skeletal muscle differentiation in mouse embryos. HIRA is a histone chaperone primarily responsible for replication-independent incorporation of H3.3 into gene bodies and regulatory elements [8]. Valenzuela et al. [9] reported that muscle fibers lacking HIRA expression exhibited hypertrophy, sarcolemmal perforation, and oxidative damage, which was concomitant with the downregulation of the expression of cellular stress-related genes. This finding suggested that H3.3 incorporation plays an important role in the susceptibility of muscle fibers to stress-induced degeneration. Furthermore, H3.3 incorporation was reportedly increased in several organs with increasing age, suggesting tissue-specific patterns of histone modifications [10]. However, the relationship between H3.3 and tissue aging in skeletal muscles remains unclear. Therefore, the present study aimed to investigate age-related changes in H3.3 and its role in the aging process of mouse skeletal muscle.

## Materials and methods

### Ethical approval and animal cares

All experimental procedures were conducted following the Guide for the Care and Use of Laboratory Animals of the Matsumoto University (Nagano, Japan). All experimental procedures were approved by the Animal Use Committee at Matsumoto University (Approval ID: 2021–4, 2022–3, 2023–3). All experimental procedures were also confirmed by ARRIVE guidelines 2.0 (https://arriveguidelines.org/arrive-guidelines). Male C57BL/6 J mice at 7, 31, 52, and 74 weeks of age were purchased from CLEA Japan (Tokyo, Japan) and used in the present study. The mice were acclimated to the experimental environment for 1 week before being used for the experiments described below. A commercial solid diet (CE-2, CLEA Japan) and water were supplied ad libitum. Temperature and humidity in the animal room were maintained at 23 °C and 40–60%, respectively, with 12:12 h light–dark cycle.

### Experimental design

In Experiment 1, the age-related changes in the histochemical properties, gene expression, and histone modifications in the tibialis anterior muscle were determined using 8-, 32-, 53-, and 75-wk-old mice (n = 3 each). To minimize the detection of changes caused by non-aging factors, the minimal number of biological replicates necessary to detect aging effects was used. In Experiment 2, responses of gene expression and histone modifications to a single bout of exercise were compared between young (8-wk-old, n = 6) and middle-aged (53-wk-old, n = 6) mice. In this experiment, 3 mice were tested for acute exercise in each age group, as the gene set used in the present study showed statistically detectable upregulation of gene expression in response to exercise with 3 biological replicates, as previously reported. [11, 12]. In Experiment 3, the effects of forced H3.3 expression in skeletal muscles on motor function were examined in young mice (8-wk-old, n = 10).

### Acute exercise (experiment 2)

The mice with the age of 8 and 53 weeks were separated into the control and exercise groups (n = 3 each). The mice in the exercise group were assigned to perform running exercise for a total of 30 min at a speed of 25 cm/s, starting at a speed of 15 cm/s, gradually increasing the speed to 20 cm/s and finally to a speed of 25 cm/s, using an animal treadmill (Panlab Harvard apparatus, Barcelona, Spain). The tibialis anterior muscles were sampled from both control and exercise groups 2 h after the end of running exercise. Muscle sampling was performed immediately after the treatment of carbon dioxide gas. Each mouse was transferred to an inhalation chamber and subsequently exposed to an increasing concentration of carbon dioxide gas. The muscle tissues were cleaned of excess fat and connective tissue. Muscle samples were frozen in liquid nitrogen and stored at − 80 °C until analysis.

### Design and administration of viral vector (experiment 3)

AAV9 was modified to encode mouse histone H3.3A (gene symbol: *H3f3a*) at the downstream of ACTA1 promoter (VectorBuilder Japan, Kanagawa, Japan). Thus, the transfection of this vector provides the skeletal muscle-specific H3.3 expression. As for the control group, the *H3f3a* sequence was replaced with untranslatable 249 bp sequence (stuffer). The mice at 8-wk-old were separated into Stuffer and H3.3 groups (n = 5 each). AAV9 vector was intravenously delivered through the tail vein under inhalation anesthesia by Isoflurane. Single shot (100 μL) was performed to administer 8 × 10^11^ vg per mouse. The tibialis anterior muscle was sampled at 32-wk-old.

To evaluate the effects of AAV9 vector administration on other organs, we also tested the comparison between CMV immediate early enhancer/promoter and ACTA1 promoter to express EGFP reporter (Fig. S1A). CMV promoter has driven the strong EGFP expression in liver, lung and kidney 2 weeks after the injection of vector, which was markedly reduced if ACTA1 promoter was used. Therefore, ACTA1 promoter was selected to use for H3.3 expression in the present study.

### Rotarod test

Motor function was tested using a rotarod (LE8205, Barcelona, Spain) in Experiment 3. Rotarod test was performed every 2 weeks until 30-wk-old. The mice were placed on the platform before starting the rotation. The rotation of platform was started at 40 rpm, and the speed of rotation was increased 4 rpm every 5 s. The latency (time) to fall was measured for each mouse. The mice were placed back on the platform immediately after fall. The test was repeated 5 times, and the mean latency to fall was calculated in each mouse.

### Immunohistochemistry

Cross-sections from the mid portions of the tibialis anterior muscles were cut at 10 µm in a cryostat (Leica Microsystems, Wetzlar, Germany) maintained at − 20 °C. The sections were fixed in 4% paraformaldehyde for 5 min, followed by blocking in 10% goat or donkey serum diluted in PBS containing 0.1% Triton X-100 (TPBS) for 20 min. Overnight incubation at 4 °C with anti-dystrophin (ab15277 or ab129996, Abcam), anti-pericentriolar material 1 (PCM1) (HPA023370, Merck), anti-type I myosin heavy chain (MyHC) (BA-D5, Developmental Studies Hybridoma Bank, Iowa City, IA), anti-type IIb MyHC (BF-F3, Developmental Studies Hybridoma Bank), and anti-type IIa MyHC (SC-71, Developmental Studies Hybridoma Bank). The antibodies were diluted at 1:100 in TPBS containing 1% bovine serum albumin (BSA). Visualization for the binding site of primary antibody was performed using Alexa Fluor 350 (A-21140, Thermo Fisher Scientific, Waltham, MA), 488(A-21121 for mouse immunoglobulin or A-21206 for rabbit immunoglobulin, Thermo Fisher Scientific), 546 (A-21045 or A-10036, Thermo Fisher Scientific) and 647 (A-31573, Thermo Fisher Scientific) diluted at 1:500 in TPBS containing 1% BSA for 1 h. The stained sections were mounted for microscopic analysis using SlowFade Gold Antifade Mountant with 4’,6-diamidino-2-phenylindole (DAPI) (S36938, Thermo Fisher Scientific) to label nuclei or antifade mounting medium (H-1000, Vector Laboratories) without nuclear labelling. The images of whole sections were incorporated into a computer using All-in-One Fluorescence Microscope system (BZ-X710, KEYENCE, Osaka, Japan). The exposure time was set constant among all sections if the pattern to use the antibodies was same.

The muscle fiber phenotype was classified by the fluorescence intensity of each MyHC isoform into type I, IIa or IIb. The fibers without labelling with these antibodies were classified as type IIx fibers [13]. All muscle fibers were targeted to analyze the fiber phenotype and size (approximately 2500 fibers per muscle section). Myonuclei were counted automatically while the green fluorescence channel labelled by the PCM1 and overlapped by the DAPI using BZ-X Analyser software (KEYENCE). The area enclosed by dystrophin (muscle fiber size) was also analyzed in all images.

### Western blotting

Total histone was extracted using Epiquik Total Histone Extraction Kit (Epigentek, Farmingdale, NY). Total histone obtained from 20 mg muscle samples were extracted in 500 µL lysis buffer packaged in the kit, centrifuged at 12,000*g* for 5 min at 4 °C, and 300 µL supernatant was collected and mixed with 90 µL balance buffer packaged in the kit. The total histone extract was further dissolved in an equal amount of 2 × SDS sample buffer (20% glycerol, 12% 2-mercaptoethanol, 4% sodium dodecyl sulfate, 100 mM Tris–HCl, and 0.05% bromophenol blue, pH 6.7) and boiled for 10 min.

Western blotting was performed as described previously [12]. The following antibodies were used to detect each protein: H3.3 (ab176840; Abcam, Cambridge, UK, 1:1000), H3.1/3.2 (61629, Active Motif, Carlsbad, CA, 1:1000), H3K4me3 (9751, Cell Signaling Technology, Danvers, MA, 1:1000), H3K9me3 (61013, Active Motif, 1:1000), H3K27me3 (9733, Cell Signaling Technology, 1:1000), H3K27ac (8173, Cell Signaling Technology, 1:1000), H3K36me3 (4909, Cell Signaling Technology, 1:1000), and total H3 (4620, Cell Signaling Technology, 1:1000). The antibody-bound protein was detected by a chemiluminescence method using ChemiDoc Touch MP (Bio-Rad, Hercules, CA). The bands were quantified using image analysis software (ImageJ) (https://imagej.net/ij/). The protein level was expressed as the integrated density of the band, which was calculated as the mean density multiplied by the band area. Finally, the integrated density was compared between the experimental groups that were applied to the same membrane.

### Gene expression

A piece of frozen muscle (10–20 mg) was homogenized in 1 ml of ISOGEN (NIPPON GENE, Toyama, Japan). RNA extraction was performed following the manufacturer’s instructions. The final pellet of RNA was resuspended in ultrapure water. Total RNA from 8-wk-old mice (n = 3) and 75-wk-old mice (n = 3) groups in Experiment 1 was combined within each group and used to construct complementary DNA (cDNA) libraries for RNA sequencing analysis. RNA-seq analysis was performed through commercial service (Novogene, Chula Vista, CA). mRNAs were enriched with oligo(dT) beads and randomly fragmented in the fragmentation buffer, followed by cDNA synthesis using random hexamers and reverse transcriptase. After the first-strand synthesis, a custom second-strand synthesis buffer (Illumina, San Diego, CA) was added along with dNTPs, RNase H, and Escherichia coli polymerase I to generate the second strand by nick translation. The final cDNA library was prepared after purification, terminal repair, A-tailing, ligation of sequencing adapters, size selection, and PCR enrichment. The NovaSeq6000 system (Illumina) was used to obtain reads of 150-bp paired ends. Approximately 40 million reads for each group were mapped to the mouse whole genome database using the hisat2 software, and the fragments per kilobase of exon per million mapped fragments (FPKM) value was calculated for the exons of all known loci. All FPKM data obtained in the RNA sequencing analysis are available in Additional file 1.

For the quantitative analysis of gene expression, SuperScript VILO Master Mix (Thermo Fisher Scientific) was used to synthesize cDNA following the manufacturer's instructions. Total RNA (800 ng) was incubated with SuperScript VILO Master Mix at 42 °C for 60 min, followed by inactivation of the enzyme at 85 °C for 5 min. Synthesized cDNA was diluted to 1:100 with ultrapure dH_2_O and stored at − 20 °C until analysis. Candidates of target genes that were up- or down-regulated by > 2 folds at 75-wk-old compared to 8-wk-old (18 upregulated and 17 downregulated genes, Additional file 1) were selected for gene expression and chromatin immunoprecipitation (ChIP) analysis, and confirmed by quantitative PCR (qPCR) using the specific primer sets (Additional file 2). Upregulated (n = 15) and downregulated (n = 14) genes were identified as aging-related genes, and targeted to analyze the gene expression and histone distributions in Experiment 1 (Fig. S2). Previously described gene set [11] was used to analyze the gene response to acute exercise in Experiment 2. These gene sets were also targeted for the analysis in Experiment 3.

### ChIP

Extraction of chromatin-rich extract and chromatin immunoprecipitation were performed as described previously [14]. Briefly, muscle segments (20–40 mg) were homogenized in cooled PBS. After centrifugation at 12,000*g*, the pellet was fixed in 1% paraformaldehyde on ice for 10 min followed by quenching in 200 mM glycine. The pellet was resuspended in lysis buffer and sonicated using a Sonifier 250 (Branson, Danbury, CT, USA). For ChIP-qPCR analysis, sonication was repeated four times, resulting in an average DNA fragment size of 500 bp. After centrifugation at 12,000*g*, the supernatant was further gel-filtrated to remove small DNA fragments and free histones, which did not form nucleosomes, and stored as the chromatin-rich extract at − 80 °C until analysis.

Chromatin-rich extracts containing equal DNA content (400 ng) were combined within each group and used for the ChIP reaction. Chromatin was reacted with anti-H3.3 (ab176840, Abcam, 1:50), anti-H3.1/3.2 (61,629, Active Motif, 1:50), anti-H3K4me3 (9751, Cell Signaling Technology, 1:50) or anti-H3K27me3 (9733, Cell Signaling Technology, 1:50) for 1 h at 4 °C, followed by a reaction with SureBeads Protein G (1,614,023, for rabbit immunoglobulin) or Protein A (1,614,013, for mouse immunoglobulin) Magnetic Beads (Bio-Rad, Hercules, CA, USA) for 30 min at 4 °C. Beads were washed and incubated with proteinase K (Takara Bio, Shiga, Japan) for 1 h at 65 °C. DNA was extracted and resuspended in Tris–EDTA buffer and stored at − 20 °C. ChIP rection using same antibody was tested twice to minimize the differences between reactions, and the yielded DNA was combined within each group. The level of input DNA contained in chromatin used for each ChIP reaction was also tested without any reactions.

### qPCR

Quantitative PCR was performed using the StepOne Real Time PCR System (Thermo Fisher Scientific). THUNDERBIRD NEXT SYBR qPCR Mix (TOYOBO, Osaka, Japan) was used for the PCR, with manufacturer-recommended dilution procedures. Sequences of primer pairs used for gene expression and ChIP-qPCR analysis are shown in Additional file 2. To analyze the histone distribution at the transcription start site (TSS), two primer pairs for ChIP-qPCR were designed at every 500 bp on 1 kbp downstream sequence from TSS. As for the exercise-related genes, previously designed primers which covered between 1 kbp upstream and downstream from TSS [11]. Since the chromatin obtained in the present study mainly contained tri-nucleosomes (500 bp peak DNA fragment size), the sites for the primer design needed to be separated by at least 500 bp for non-overlapping analysis.

Quantification of the qPCR results was performed by normalizing to the cycle threshold (Ct) of the target amplification, with *Gapdh* or *Rpl31* mRNA as the internal control for gene expression assay or with the Ct of respective input ChIP-qPCR (% input). The % inputs obtained from each locus were averaged for each mouse. For the presentation of ChIP data, the % input was further normalized by the average of all groups in each gene.

### Statistical analysis

Statistical analysis was performed using BellCurve for Excel (Social Survey Research Information, Tokyo, Japan). Significant difference was examined by one-way (Experiment 1) or two-way (Experiment 2) ANOVA followed by Scheffe’s post hoc test. Student’s unpaired* t* test was used to compare the two groups (Experiment 3). Differences were considered significant at p < 0.05. Pearson correlation was used to test the significance of the strength and direction of association between two factors (Experiment 1 and 3).

## Results

### Age-related changes in mouse tibialis anterior muscle

**Fig. 1:**
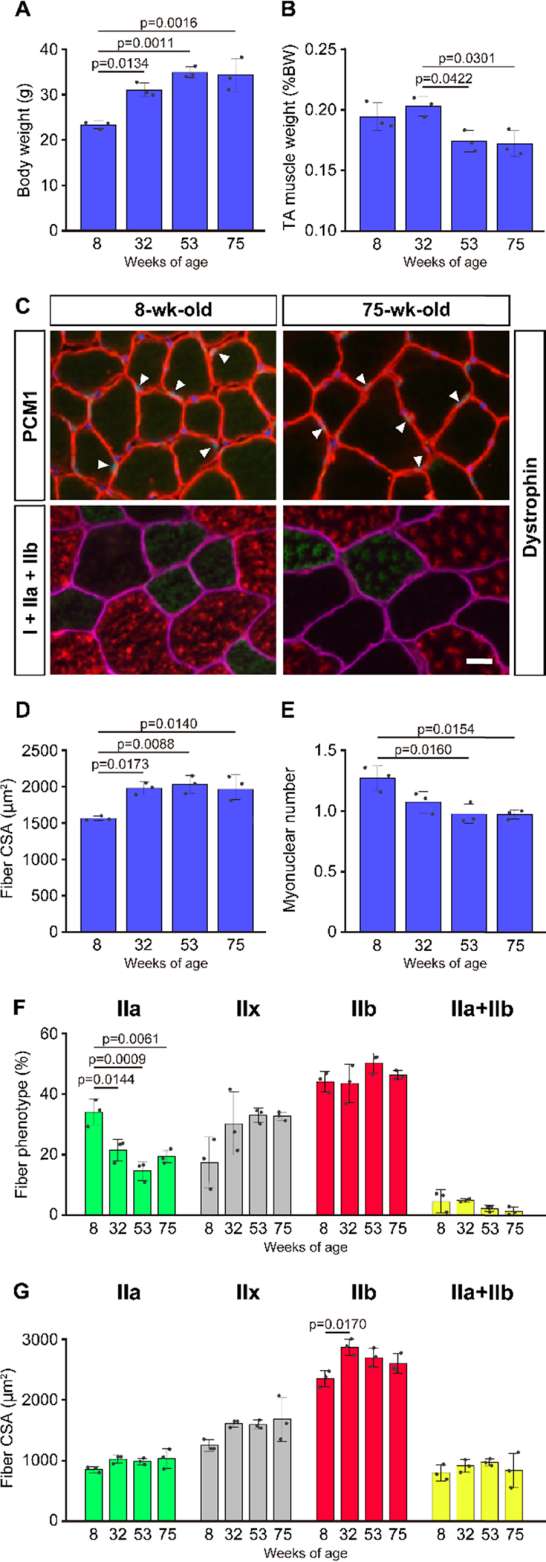
Age-related changes in histochemical properties of muscle fibers. Body weight (**A**) and tibialis anterior muscle weight relative to body weight (**B**) at 8, 32, 53, and 75 weeks of age. (**C**) Typical stained images of PCM1 (green) + dystrophin (red) + DAPI (blue) (upper panels), and type I (blue) + IIa (green) + IIb (red) myosin heavy chains + dystrophin (pink) (lower panels) at 8 and 75 weeks of age. Myonuclei are indicated by arrowheads. Scale bar = 10 µm. Mean fiber cross-sectional area (CSA) (**D**), myonuclear number per fiber (**E**), distribution of fiber phenotype (**F**), and CSA of each fiber phenotype (**G**) at 8, 32, 53, and 75 weeks of age. Data are presented as the mean ± standard deviation (SD); n = 3 in each group

First, we examined morphological changes in tibialis anterior muscle fibers as a result of aging using 8-, 32-, 53-, and 75-wk-old mice. Body weight of 32-wk-old mice was significantly higher than that of 8-wk-old mice (Fig. 1A), and minor gains in body weight were observed in the 53- and 75-wk-old mice. Compared to that in 8-wk-old mice, the relative weight of the tibialis anterior was maintained in 32-wk-old mice; however, the same was significantly decreased in 53-wk-old mice (Fig. 1B). With respect to muscle fiber size, compared to 8-wk-old mice, muscle fiber size increased significantly and reached a peak at 32 weeks (Fig. 1C and D). The number of myonuclei decreased significantly after 53 weeks of age, compared to that at 8 weeks of age (Fig. 1C and E). While type I fibers were not observed in any age group, the number of type IIa fibers significantly decreased after 32 weeks of age (Fig. 1F). In contrast, other fiber types (i.e., IIx, IIb, and IIa + IIb) were not affected by aging (Fig. 1F). Fiber CSA of type IIb fibers significantly increased between 8- and 32-wk-old, whereas no significant differences were seen in fiber CSA of type IIb fibers between 8- and 53- or 75-wk-old (Fig. 1G). Fiber CSA of other fiber types (i.e., IIa, IIx, and IIa + IIb) was not changed with age.

**Fig. 2:**
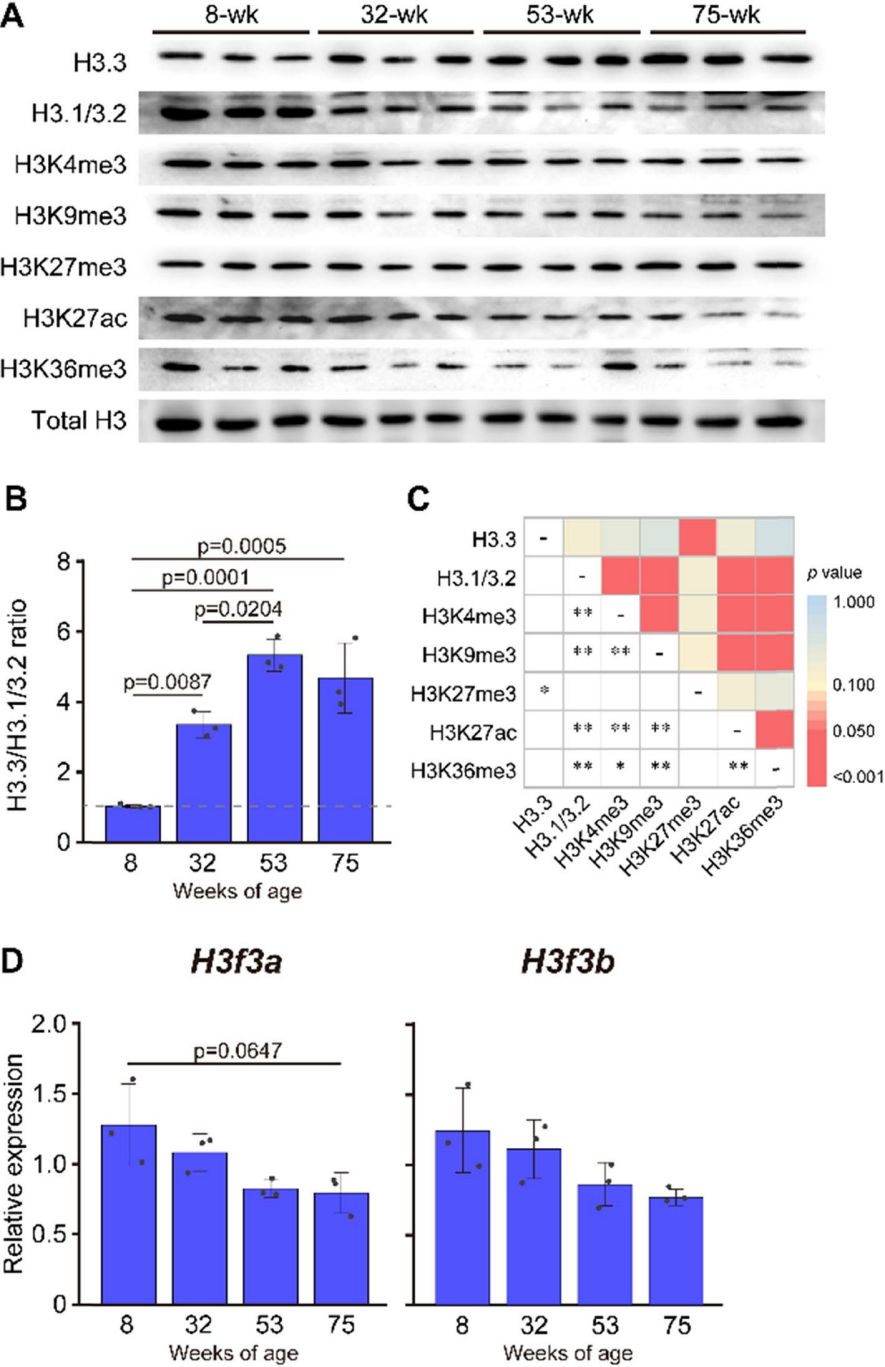
Age-related changes in histone modifications in skeletal muscle genes. (**A**) Western blot analysis of H3.3, H3.1/3.2, H3K4me3, H3K9me3, H3K27me3, H3K27ac, H3K36me3, and total H3 levels at 8, 32, 53, and 75 weeks of age. (**B**) H3.3 to H3.1/3.2 ratio at 8, 32, 53, and 75 weeks of age. (**C**) Heat map showing the *p* values obtained using Pearson correlation between various histone modifications. *p < 0.05. **p < 0.01. See Fig. S3 for more precise data plots for the correlation of H3.3 or H3.1/32 with various histone modifications. (**D**) *H3f3a* and *H3f3b* mRNA expression at 8, 32, 53, and 75 weeks of age. Data are represented as the relative expression of target mRNA to *Gapdh* mRNA. (**B**), (**D**) Data are presented as the mean ± SD; n = 3 in each group

Next, we assessed age-related changes in histone expression in tibialis anterior muscle fibers. H3.3 levels, relative to the canonical variants H3.1/3.2, were significantly increased in 32-wk-old mice compared to that in 8-wk-old mice, and the levels peaked at 53 weeks of age (Fig. 2A and B). Levels of all histone modifications, except for H3K27me3, decreased with age (Fig. S3A). H3.3 levels were significantly correlated with H3K27me3 levels, whereas H3.1/3.2 levels were significantly correlated with H3K4me3, H3K9me3, H3K27ac, and H3K36me3 levels (Fig. 2C and S3B). H3.3 is encoded by two genes, *H3f3a* and *H3f3b*. *H3f3a* and *H3f3b* mRNA expression was found to be downregulated with age, with levels 38% (p = 0.0647) and 37% (p = 0.1209) lower, respectively, in 75-wk-old mice compared to that in 8-wk-old mice (Fig. 2D).

**Fig. 3:**
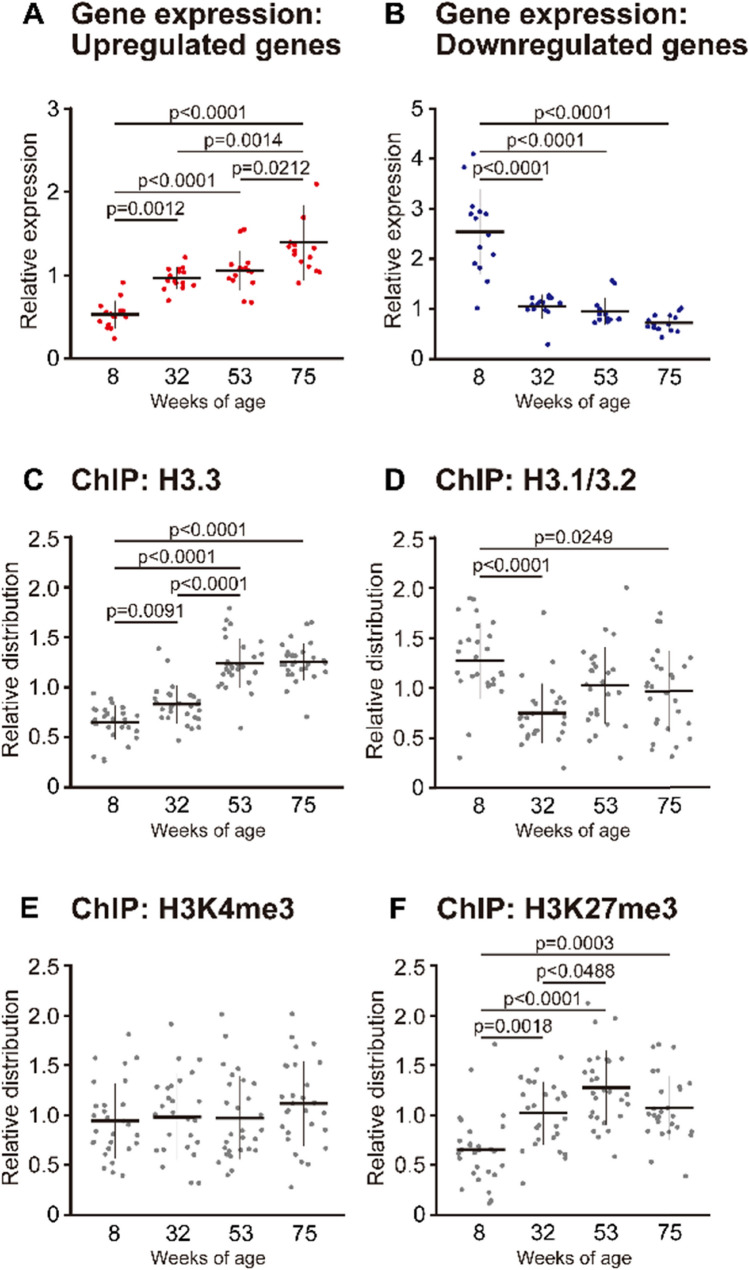
Age-related changes in gene expression and histone distribution of skeletal muscle genes. Genes that were upregulated (n = 15) (**A**) and downregulated (n = 14) (**B**) at 8, 32, 53, and 75 weeks of age. Data are presented as the relative expression of target mRNA to *Rpl31* mRNA. Target genes were selected by RNA sequencing analysis. Significance of age-related change (8- vs. 75-wk-old) was confirmed in each gene by qPCR analysis (Fig. S2). Distribution of H3.3 (**C**), H3.1/3.2 (**D**), H3K4me3 (**E**), and H3K27me3 (**F**) at 8, 32, 53, and 75 weeks of age was assessed by ChIP analysis. Values are normalized to the average in each gene being taken as 1. Data are presented as the mean ± SD on the dot plots

Furthermore, we assessed age-related changes in the expression of genes that were differentially expressed with age. The expression of the upregulated genes increased significantly (+ 45%) at 32 weeks of age compared to that at 8 weeks of age (Fig. 3A). A further increase was observed at 75 weeks of age compared to that at 32 weeks of age (+ 31%, p < 0.05). On the other hand, the expression of downregulated genes decreased significantly (− 59%) at 32 weeks of age compared to that at 8 weeks of age; however, no change in expression was noted between 32- and 75-wk-old mice (Fig. 3B). The results of the distributions of histones on the upregulated and downregulated genes was presented together, as both showed the same trend. The distribution of H3.3 increased significantly (+ 22%) at 32 weeks of age compared to that at 8 weeks of age (Fig. 3C). The distribution of H3.3 further increased and peaked at 53 weeks of age compared to that at 32 weeks of age (+ 33%, p < 0.05). The distribution of H3.1/3.2 decreased significantly (− 40%) at 32 weeks of age compared to that at 8 weeks of age, and was maintained at low levels at 75 weeks of age (Fig. 3D). Aging did not affect H3K4me3 distribution (Fig. 3E). On the other hand, H3K27me3 distribution followed the same trend as that observed for H3.3 (Fig. 3F).

### Responses to acute exercise

**Fig. 4:**
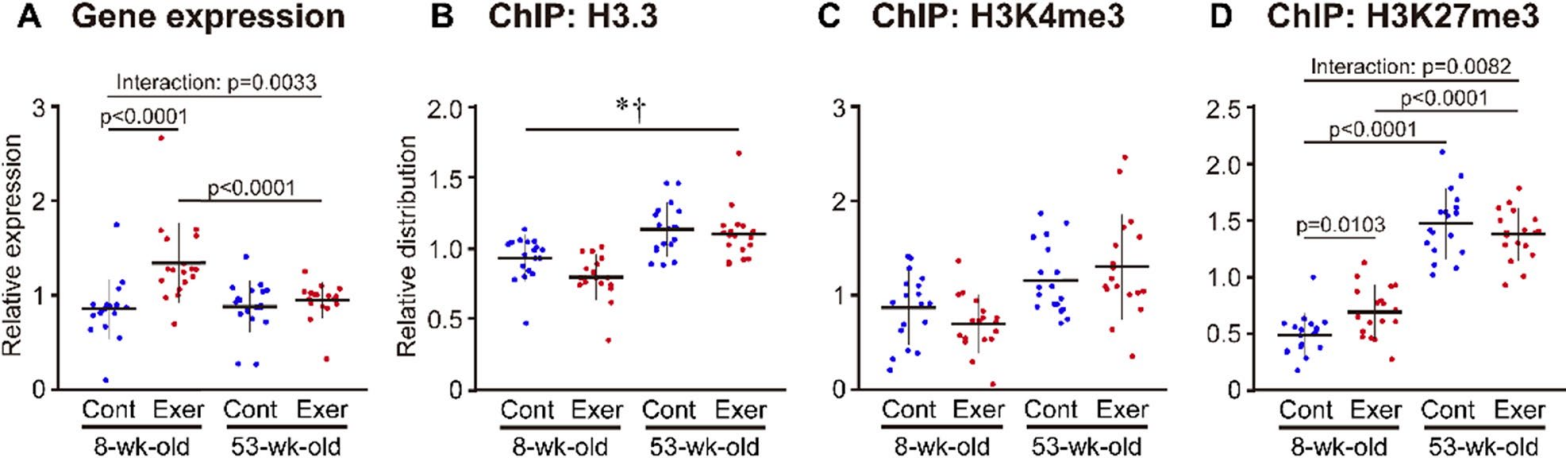
Effects of acute exercise on gene expression and histone modifications of skeletal muscle genes in 8- and 53-wk-old mice. The expression of previously identified 19 exercise-related genes [11] was analyzed (**A**), alongside the distribution of H3.3 (**B**), H3K4me3 (**C**) and H3K27me3 (**D**) at these target loci using ChIP analysis. (**A**) Data are presented as the relative expression of target mRNA to *Gapdh* mRNA. (**A-D**) Values are normalized to the average in each gene being taken as 1. Data are presented as the mean ± SD on the dot plots. *P* values resulted from Scheffe’s post hoc test are shown in the figures, if a significant interaction was detected by two-way ANOVA. * and ^†^*p* < 0.05 in main effect of age and exercise, respectively

Next, we assessed the effect of acute exercise on epigenetic regulation of skeletal muscle function of 8- and 53-wk-old mice. We found that following acute exercise, the expression of the target genes was significantly upregulated (+ 57%) in 8-wk-old mice compared to that in the control group (Fig. 4A). On the other hand, the same was not observed in 53-wk-old mice following acute exercise. With respect to H3.3 distribution at target loci, a main effect of exercise (p = 0.0352) was observed, but a stronger main effect of age (p < 0.0001) was observed between 8- and 53-wk-old mice (Fig. 4B). H3K27me3 distribution increased (3 folds, p < 0.05) in 53-wk-old mice compared to that in 8-wk-old mice (Fig. 4D). Acute exercise stimulated H3K27me3 accumulation at target loci in 8-wk-old mice compared to that in the control group (+ 43%, p < 0.05), whereas H3K27me3 distribution was not affected by acute exercise in 53-wk-old mice. On the other hand, H3K4me3 distribution was similar between 8- and 53-wk-old mice and was not affected by acute exercise (Fig. 4C).

### Effects of forced H3.3 expression

**Fig. 5:**
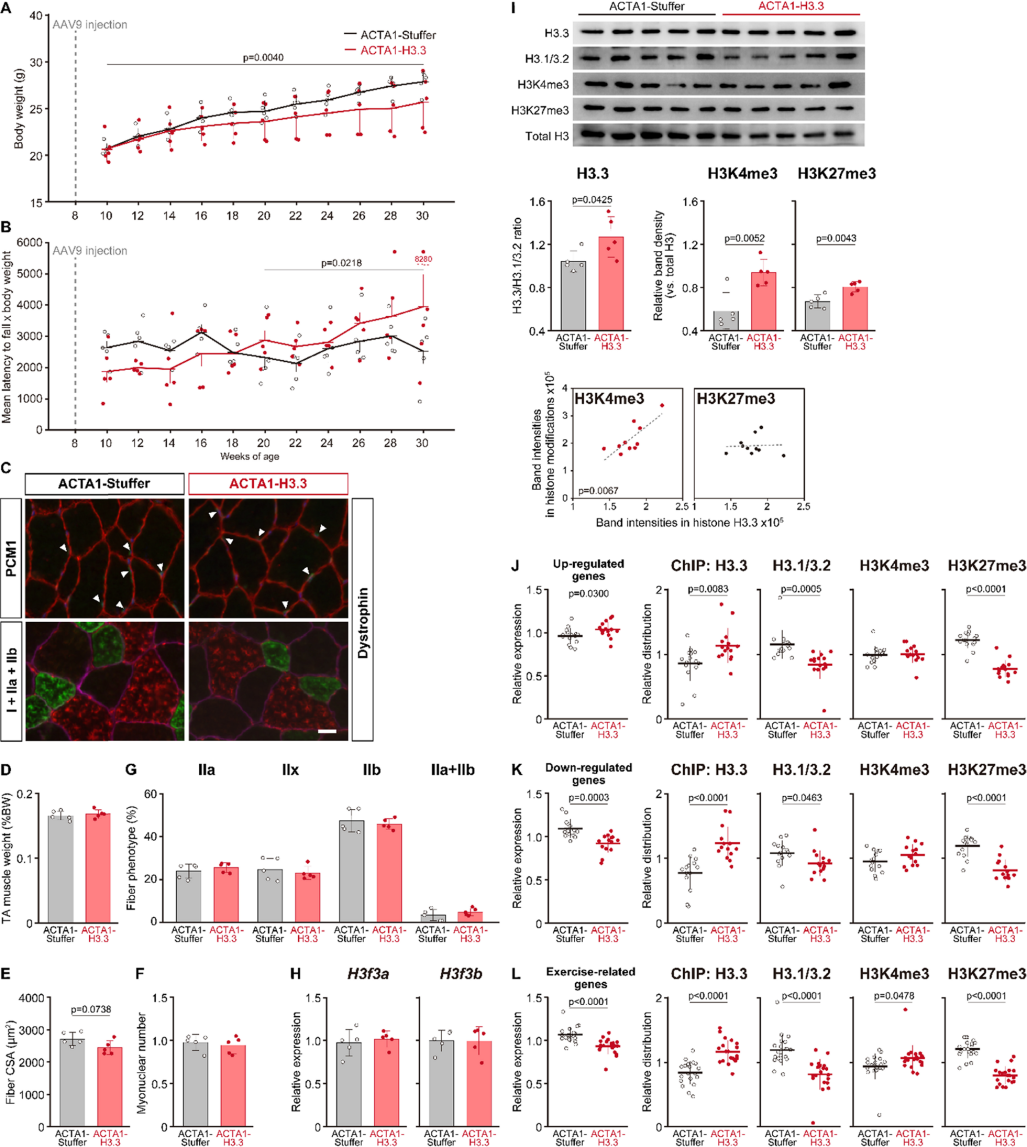
Effects of forced H3.3 expression on skeletal muscle of 8-wk-old mice. Age-related changes in body weight (**A**) and latency to fall in rotarod test (**B**) in mice with or without forced H3.3 expression. The values (time) obtained in the rotarod test were averaged in five trials and were multiplied by the respective body weight (g) to calculate the integrated values. To examine the consequence of changes in the period, the maximum length of the continuous time points from the end of experiment (30-wk-old) was selected for Student’s unpaired *t* test. (**C**) Typical stained images of PCM1 (green) + dystrophin (red) + DAPI (blue) (upper panels), and type I (blue) + IIa (green) + IIb (red) myosin heavy chains + dystrophin (pink) (lower panels) in the Stuffer (left) and H3.3 (right) groups. Myonuclei are indicated by arrowheads. Scale bar = 10 µm. (**D**) Tibialis anterior muscle weight relative to body weight. (**E**) Mean fiber cross-sectional area (CSA). (**F**) Myonuclear number per fiber. (**G**) Distribution of fiber phenotype. (**H**) *H3f3a* and *H3f3b* mRNA expression. Data are represented as the relative expression of target mRNA to *Gapdh* mRNA. (**I**) Western blot analysis of H3.3, H3.1/3.2, H3K4me3, H3K27me3, total H3 levels (upper images), and the quantification of each protein (bar graphs). H3K4me3 and H3K27me3 levels were normalized to the respective total H3 levels. X–Y plots show the Pearson correlation between H3.3 and H3K4me3 or H3K27me3. (**J**–**L**) Changes in gene expression and the distribution of H3.3, H3.1/3.2, H3K4me3 and H3K27me3 at the upregulated (**J**), downregulated (**K**), and exercise-related (**L**) genes. Data were presented as the relative expression of target mRNA to *Rpl31* mRNA. Values are normalized to the average in each gene being taken as 1. Data are presented as the mean ± SD on the dot plots. Biological replicates: n = 5 in each group. Bar graph present data from biological replicates, and dot plots present gene-related data

To investigate the role of age-related increases in H3.3 distribution on skeletal muscle genes, we assessed the effect of forced H3.3 expression in 8-wk-old mice, as H3.3 was maintained at low levels in this age group. Age-associated increase in body weight was significantly inhibited in the H3.3 group compared to that in the Stuffer group (Fig. 5A). The integrated score of the rotarod test, which was expressed by multiplying the mean latency to fall by the body weight, was significantly higher in the H3.3 group than that in the Stuffer group starting at 20 weeks of age (Fig. 5B). However, no significant changes were observed in the muscle weight, fiber size, myonuclear number, or fiber phenotype of the tibialis anterior muscle after treatment with the ACTA1-H3.3 vector (Fig. 5C–G).

Although *H3f3a* and *H3f3b* mRNA expression was not affected by treatment with the ACTA1-H3.3 vector (Fig. 5H), H3.3 protein levels were significantly increased (+ 18%) in the H3.3 group compared to that in the Stuffer group (Fig. 5I). Total levels of H3K4me3 and H3K27me3 were increased following treatment with ACTA1-H3.3 vector in the H3.3 group compared to that in the Stuffer group (Fig. 5I). The level of H3.3 significantly correlated with the level of H3K4me3, whereas no correlation was observed between H3.3 and H3K27me3 levels (Fig. 5I).

Upregulated and downregulated genes identified in Experiment 1 and exercise-related genes used in Experiment 2 were assessed to analyze the effect of forced H3.3 expression on gene expression and histone distribution. The expression of downregulated and exercise-related genes was significantly decreased (− 16% and − 13%, respectively) in the H3.3 group compared to that in the Stuffer group, whereas the expression of upregulated genes was significantly increased (+ 7%) in the H3.3 group than compared to that in the Stuffer group (Fig. 5J–L). The results of ChIP-qPCR analysis showed an increase in H3.3 distribution and a decrease in H3.1/3.2 and H3K27me3 distribution at all targeted loci (Fig. 5J–L). Additionally, the distribution of H3K4me3 was significantly increased at exercise-related loci.

## Discussion

### Relationship between skeletal muscle aging and H3.3

Tibialis anterior muscle is one of the muscles that exhibits significant decline in muscle mass with age [15]. Therefore, in the present study, we used the tibialis anterior muscle. It was reported that the tibialis anterior muscle weight and fiber size were significantly decreased in 2-yr-old mice compared to adult mice aged 2–6 months [16–18]. We observed the onset of abnormalities in muscle mass relative to body weight at 53 weeks of age (Fig. 1B), indicating that muscle growth decelerated against the rate of body mass gain. The data obtained in the present study also agree with previous studies [16, 19] demonstrating that fiber CSA in the tibialis anterior muscle was maintained in 13-month-old mice and did not significantly decline by 21–24 months, remaining comparable to that in adult mice aged 3–6 months. These observations suggest that the tibialis anterior muscle in middle-aged to old mice (53- and 75-wk-old in the present study) is in a pre-atrophic state. Given that myonuclear number significantly decreased at 53 weeks of age (Fig. 1E), the loss of myonuclei potentially inhibited additional hypertrophy of muscle fibers. Generally, muscle fiber hypertrophy is associated with the accretion of myonuclei. However, it has been reported that a transgenic model inducing a conditional loss of satellite cells in skeletal muscle showed successful fiber hypertrophy without an increase in myonuclei after the ablation of synergists, indicating that an expansion of the myonuclear domain can compensate for the lack of myonuclear accretion during hypertrophy [20, 21]. Since other factors, such as oxidative stress [22], abnormal autophagy [23], and neuromuscular junction disorders [24], also play key roles in the progression of skeletal muscle senescence, the onset of muscle mass wasting might be caused by a combination of these factors.

Tibialis anterior muscle is predominantly composed of type II fibers. It was noted that the number of type IIa fibers were significantly decreased after 32-wk-old compared to 8-wk-old (Fig. 1F). Previous studies [16–18] also demonstrated a decrease in type IIa fibers and an increase in type IIb fibers in mouse tibialis anterior muscle during aging, supporting the results in the present study. Considering the fiber size and distribution of type IIb fibers in the tibialis anterior muscle, the size of type IIb fibers might be most reflective of total muscle mass, which peaked at 32 weeks of age. No significant differences were seen in the CSA of type IIb fibers between 8- and 53- or 75-wk-old (Fig. 1G), suggesting a trend towards decreasing fiber size with age. Therefore, age-related changes in the mouse tibialis anterior muscle between 8 and 75 weeks old were characterized by a decrease in type IIa fibers and inhibited growth of type IIb fibers. These phenomena might also cause changes in histone components and modifications that discussed below.

The results of the present study demonstrated that H3.3 accumulation increased with age in mouse tibialis anterior muscle (Fig. 2B). Interestingly, *H3f3a* and *H3f3b* mRNA expression was downregulated or unchanged with age (Fig. 2D), indicating that age-related H3.3 accumulation was not caused by transcriptional activation. H3.3 incorporated into nucleosomes might be capable of escaping breakdown. Since H3.3 is incorporated into transcriptionally active sites [25], it is speculated that H3.3 persists as a component of nucleosomes through the exchange from H3.1/3.2 to H3.3. Age-related increase in H3.3 accumulation was significantly correlated with H3K27me3 levels (Fig. 2C). This trend was also observed at the loci that were transcriptionally upregulated and downregulated with aging (Fig. 3). H3K27me3 is a heterochromatin-associated histone modification that represses gene transcription [26]. With respect to age-related changes in gene expression, the decreased expression of downregulated genes preceded the increased expression of the upregulated genes during aging (Fig. 3). These results suggest that chromatin was transformed into a transcriptionally repressive conformation in association with H3.3 incorporation during aging. However, the results in the upregulated loci suggested that transcriptional activation by transcriptional factors was the primary mechanism regulating the rate of gene transcription, although the epigenetic mechanism is closely related to responsiveness to transcriptional activation.

We also determined the accumulation of H3.3 and its correlation with histone modifications in soleus and masseter muscles of the mice used in Experiment 1 (Fig. S4 and S5). Similar to the tibialis anterior muscle, the level of H3.3 significantly increased with age in both the soleus and masseter muscles. Soleus and masseter muscles are reportedly less declined with age in terms of the muscle weight [15], suggesting that H3.3 accumulation positively regulates the muscle mass. Furthermore, the patterns of histone modifications were completely different among the types of skeletal muscles (Fig. S4 and S5). In mice, soleus muscle is predominantly composed of type I and IIa fibers [17], whereas masseter muscle contains more type IIx fibers compared to tibialis anterior muscle [27]. The distribution of fiber phenotype and the muscular activity patterns might be the cause for varying the histone modifications to H3.3.

Gene responsiveness to exercise is an important function of skeletal muscles. Shimizu and Kawano [12] demonstrated that both H3K27me3 and H3K4me3 were prevalent at loci that were transcriptionally responsive to acute exercise in mouse tibialis anterior muscle. They also showed that acute exercise further stimulates the accumulation of these modifications at transcriptionally upregulated loci, suggesting that H3K27me3 acts as an active modification associated with H3K4me3. In the present study, 53-wk-old mice failed to upregulate gene expression in response to acute exercise, whereas 8-wk-old mice responded successfully to acute exercise (Fig. 4). The lower ratio of H3K4me3 to H3K27me3 at rest might be one of the causes underlying the repression of gene response to acute exercise in 53-wk-old mice. These findings suggest that age-related H3.3 accumulation causes epigenetic alterations that repress gene transcription in skeletal muscle.

### Function of H3.3 in skeletal muscle

Age-related changes in epigenetics were partially simulated by forced H3.3 expression in 8-wk-old mice. At 32 weeks of age (end of Experiment 3), H3.3 levels were successfully increased in the tibialis anterior muscle of mice that were administered ACTA1-H3.3 vector (Fig. 5I). However, no increase in *H3f3a* mRNA expression was observed at this age (Fig. 5H). We also confirmed that the viral vector persisted in the tibialis anterior muscle at 32-wk-old (end of Experiment 3) (Fig. S6). Although the reason for this phenomenon remains unclear, it is possible that the induction of gene transcription by the ACTA1 promoter was weak. Since the observed age-related increase in H3.3 levels were not associated with upregulation of mRNA expression, we speculated that mechanisms that mediate immediate degradation of mRNA underlie the observed phenomenon. Therefore, the persistence of transcriptional activity was confirmed at 32 weeks after administration of the GFP-expressing AAV9 vector at 8 weeks of age (Fig. S1B). GFP levels at 32 weeks of age (24 weeks after injection) were significantly enhanced (a sixfold increase) compared to that at 10 weeks of age (2 weeks after injection) in the tibialis anterior muscle. Similar effects were found in the soleus, lateral gastrocnemius and masseter muscles (Fig. S1B), indicating that the production of protein originating from the viral vector actually increased in skeletal muscles throughout the body 24 weeks after injection. Furthermore, to determine the level of transgene induction, HA-tagged H3.3 was expressed under the control of the ACTA1 promoter via intramuscular injection of AAV9 vector into the tibialis anterior muscle (Fig. S7). The injection of viral vector significantly upregulated the gene expression of *H3f3a*. Although the HA tag was successfully detected in the muscle injected with the viral vector, the protein level of H3.3 was not affected. These results suggest that the ACTA1 promoter exhibits weak transcriptional activity with respect to endogenous H3.3 in the tibialis anterior muscle. We additionally assessed the effect of administration of AAV9 vector in 23-wk-old mice, and the tibialis anterior muscles were sampled at 53 and 75 weeks of age. However, H3.3 levels were not enhanced at either time point (Fig. S8), suggesting that the viral vector itself and the promoter activity were not strong enough to induce H3.3 expression in the skeletal muscle of adult mice in which the onset of H3.3 accumulation had already occurred.

Forced H3.3 expression in the skeletal muscle resulted in gradual improvement in motor function (Fig. 5B), indicating that the enhancement of H3.3 plays a positive role in skeletal muscle function. Shoji et al. [28] reported that the latency to fall in the rotarod test gradually decreased with age, and this decline in motor function might be explained by an increase in body weight. The results obtained in the present study showed a significant suppression of age-related body mass gain in the H3.3 group (Fig. 5A). Therefore, the data are presented as an integrated value that multiplied the latency by body weight. The fiber CSA of the tibialis anterior muscle in the H3.3 group was slightly smaller (p = 0.0738) than that in the Stuffer group, which was associated with inhibited body mass gain (Fig. 5E). No changes were observed in the fiber phenotype distribution due to forced H3.3 expression (Fig. 5G). Therefore, the improved motor function in the H3.3 group might not be attributed to morphological changes in the tibialis anterior muscle fibers. Although the precise parameters were not determined in the present study, metabolic changes leading to the loss of body mass might be influenced by enhanced H3.3 expression in skeletal muscles.

Gene expression showed a similar change between forced H3.3 expression and aging models (Figs. 3A, B, 5J and K). Given that forced H3.3 expression enhanced motor function, age-related changes in gene expression might not be the trigger for the decline in skeletal muscle function. The magnitude of change in gene expression by forced H3.3 expression was more prominent in the downregulated genes, suggesting that H3.3 incorporation into nucleosomes plays a repressive role in gene transcription. However, forced H3.3 expression in 8-wk-old mice resulted in significant downregulation and dissociation of H3K27me3 at target loci (Figs. 5J–L). This suggests that H3K27me3 modification is not always associated with H3.3, and that the upstream pathways that modify H3K27 are critical factors for these modifications. H3K4me3 showed a significant correlation with H3.3 increase by forced expression (Fig. 5I). Indeed, ChIP analysis demonstrated that the distribution of H3K4me3 was unchanged or upregulated at the target loci (Fig. 5J–L). Although the precise mechanisms regulating gene transcription in an epigenetic manner are unclear, an altered balance between H3K4me3 and H3K27me3 might affect the regulation of gene transcription.

### Hypothetical model

**Fig. 6:**
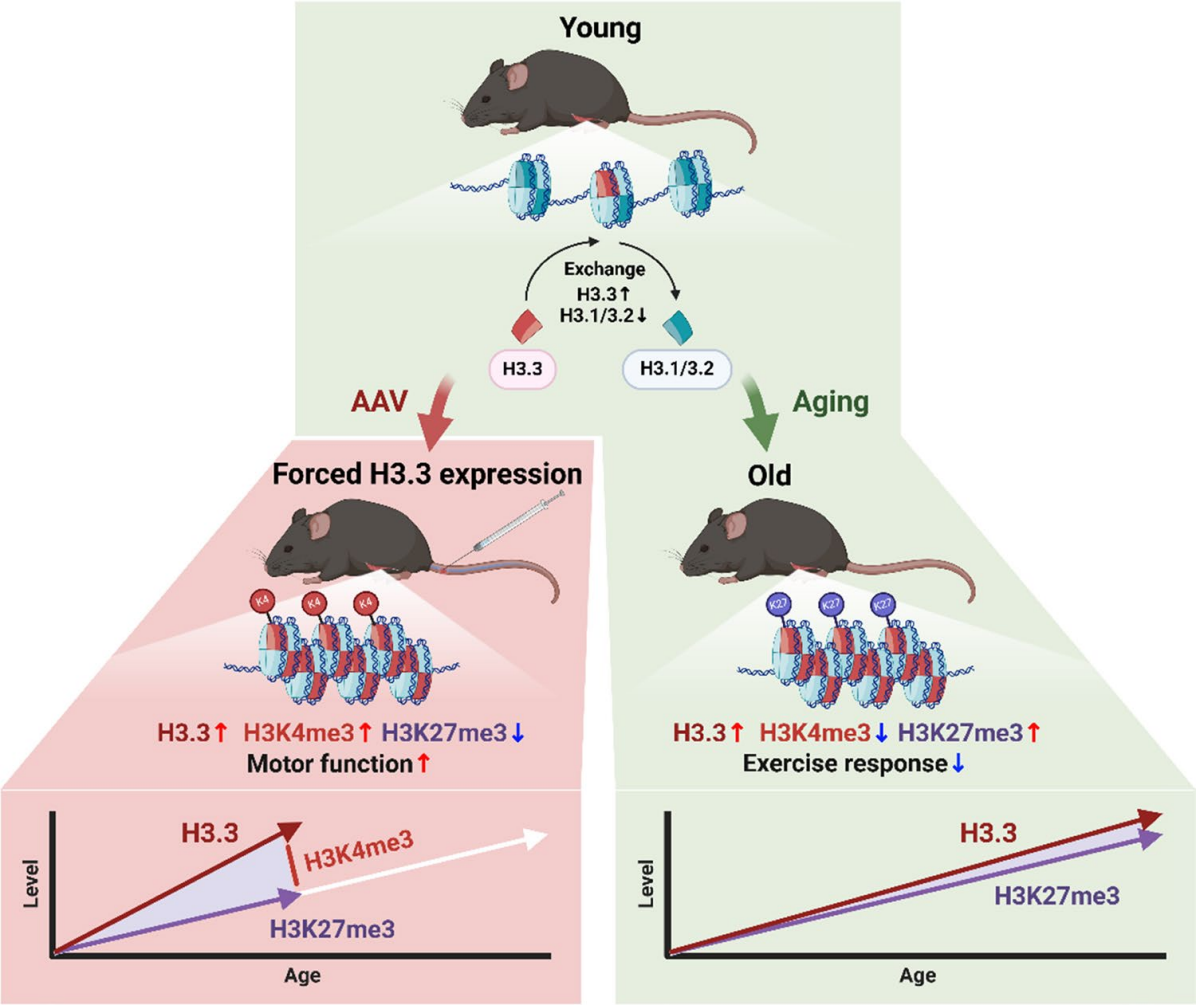
Hypothetical model illustrating the role of H3.3 accumulation in skeletal muscle. The right panel indicates a normal aging process of skeletal muscle, as suggested by the results in Experiments 1 and 2. The left panel shows the effects of forced H3.3 expression in the skeletal muscle of young mice. Note that H3.3 accumulation is a common result between aging and forced expression, but the role of the H3.3 increase in skeletal muscle function differs due to its modification pattern. See the Hypothetical model section for more details

Based on the results of the present study, a hypothetical model for the role of H3.3 in the tibialis anterior muscle of mice during aging is suggested in Fig. 6. Canonical histone H3.1/3.2 is replaced with non-canonical histone H3.3 with age in skeletal muscles. Age-related alterations in tibialis anterior muscle epigenetics generally result in a shift toward a transcriptionally repressive state due to low levels of H3K4me3 and high levels of H3K27me3 (right panel in Fig. 6). In young mice (8-wk-old), forced H3.3 expression using an AAV vector resulted in an increase in H3.3 that was modified by H3K4me3, but not H3K27me3 (left panel in Fig. 6). Although the relationship between transcriptional regulation and the pattern of histone modifications remains unclear, the histone modification pattern characterized by high H3K4me3 relative to H3K27me3 might play a positive role in motor function. These differing effects of H3.3 accumulation between aging and forced expression models suggest that the activation of H3K27me3 modifiers is age-dependent and competes with H3K4me3.

## Conclusions

The present study investigated age-related changes in H3.3 and its role in the aging process of mouse tibialis anterior muscle. Experiment 1 showed that H3.3 relative to H3.1/3.2 increased with age and significantly correlated with H3K27me3. An acute exercise test in Experiment 2 revealed reduced gene responsiveness to exercise in the tibialis anterior muscle of middle-aged mice, where H3.3 and H3K27me3 were prevalent at exercise-responsive loci. Experiment 3 demonstrated that forced H3.3 expression in the skeletal muscles of young mice resulted in a gradual improvement in motor function, as assessed by a rotarod test. These results indicate that H3.3 accumulation alters the chromatin into a repressive state, although H3.3 accumulation itself plays a positive role in motor function. It is also suggested that the function of H3.3 is closely related to its modification pattern.

## Supplementary Information

Additional file 1. Full data obtained in RNA sequencing analysis. Candidates of target genes for analysis are also shown in the separated pages.

Additional file 2. The sequences of primers used in the present study.

Additional file 3. All datasets used for figures.

Additional file 4. Figure S1–S8. Supporting figures and their legends.

## Data Availability

All data generated or analyzed during this study are included in this published article and its supplementary information files.

## Abbreviations

H3K27me3: Histone H3 trimethylated at lysine 27
EZH2: Enhancer of zeste homolog 2
H3K27ac: Histone H3 acetylated at lysine 27
H3K4me3: Histone H3 trimethylated at lysine 4
HIRA: Histone cell cycle regulator
AAV9: Adeno-associated virus (serotype 9)
ACTA1: Human skeletal muscle actine alpha 1
CMV: Human cytomegalovirus
EGFP: Enhanced green fluorescent protein

## References

[1] Wiedmer P, Jung T, Castro JP, Pomatto LCD, Sun PY, Davies KJA, Grune T (2021) Sarcopenia—molecular mechanisms and open questions. Ageing Res Rev 65:101200. 10.1016/j.arr.2020.101200 33130247 10.1016/j.arr.2020.101200

[2] Lee MM, Jebb SA, Oke J, Piernas C (2020) Reference values for skeletal muscle mass and fat mass measured by bioelectrical impedance in 390 565 UK adults. J Cachexia Sarcopenia Muscle 11:487–496. 10.1002/jcsm.12523 31943835 10.1002/jcsm.12523 PMC7113534

[3] Kelly TL, Wilson KE, Heymsfield SB (2009) Dual energy X-Ray absorptiometry body composition reference values from NHANES. PLoS One 4:e7038. 10.1371/journal.pone.0007038 19753111 10.1371/journal.pone.0007038 PMC2737140

[4] Zykovich A, Hubbard A, Flynn JM, Tarnopolsky M, Fraga MF, Kerksick C, Ogborn D, MacNeil L, Mooney SD, Melov S (2014) Genome-wide DNA methylation changes with age in disease-free human skeletal muscle. Aging Cell 13:360–366. 10.1111/acel.12180 24304487 10.1111/acel.12180 PMC3954952

[5] Voisin S, Seale K, Jacques M, Landen S, Harvey NR, Haupt LM, Griffiths LR, Ashton KJ, Coffey VG, Thompson JM, Doering TM, Lindholm ME, Walsh C, Davison G, Irwin R, McBride C, Hansson O, Asplund O, Heikkinen AE, Piirila P, Pietilainen KH, Ollikainen M, Blocquiaux S, Thomis M, Coletta DK, Sharples AP, Eynon N (2024) Exercise is associated with younger methylome and transcriptome profiles in human skeletal muscle. Aging Cell 23:e13859. 10.1111/acel.13859 37128843 10.1111/acel.13859 PMC10776126

[6] Antoun E, Garratt ES, Taddei A, Burton MA, Barton SJ, Titcombe P, Westbury LD, Baczynska A, Migliavacca E, Feige JN, Sydall HE, Dennison E, Dodds R, Roberts HC, Richardson P, Sayer AA, Shaw S, Cooper C, Holbrook JD, Patel HP, Godfrey KM, Lillycrop KA, EpiGen Global Research C (2022) Epigenome-wide association study of sarcopenia: findings from the Hertfordshire Sarcopenia Study (HSS). J Cachexia Sarcopenia Muscle. 13:240–253. 10.1002/jcsm.12876 34862756 10.1002/jcsm.12876 PMC8818655

[7] Harada A, Maehara K, Sato Y, Konno D, Tachibana T, Kimura H, Ohkawa Y (2015) Incorporation of histone H3.1 suppresses the lineage potential of skeletal muscle. Nucleic Acids Res 43:775–786. 10.1093/nar/gku1346 25539924 10.1093/nar/gku1346 PMC4333396

[8] Ray-Gallet D, Woolfe A, Vassias I, Pellentz C, Lacoste N, Puri A, Schultz DC, Pchelintsev NA, Adams PD, Jansen LE, Almouzni G (2011) Dynamics of histone H3 deposition in vivo reveal a nucleosome gap-filling mechanism for H3.3 to maintain chromatin integrity. Mol Cell 44:928–941. 10.1016/j.molcel.2011.12.006 22195966 10.1016/j.molcel.2011.12.006

[9] Valenzuela N, Soibam B, Li L, Wang J, Byers LA, Liu Y, Schwartz RJ, Stewart MD (2017) HIRA deficiency in muscle fibers causes hypertrophy and susceptibility to oxidative stress. J Cell Sci 130:2551–2563. 10.1242/jcs.200642 28600325 10.1242/jcs.200642

[10] Tvardovskiy A, Schwammle V, Kempf SJ, Rogowska-Wrzesinska A, Jensen ON (2017) Accumulation of histone variant H3.3 with age is associated with profound changes in the histone methylation landscape. Nucleic Acids Res 45:9272–9289. 10.1093/nar/gkx696 28934504 10.1093/nar/gkx696 PMC5766163

[11] Ohsawa I, Kawano F (2021) Chronic exercise training activates histone turnover in mouse skeletal muscle fibers. FASEB J 35:e21453. 10.1096/fj.202002027RR 33749947 10.1096/fj.202002027RR

[12] Shimizu J, Kawano F (2022) Exercise-induced histone H3 trimethylation at lysine 27 facilitates the adaptation of skeletal muscle to exercise in mice. J Physiol 600:3331–3353. 10.1113/JP282917 35666835 10.1113/JP282917

[13] Sandona D, Desaphy JF, Camerino GM, Bianchini E, Ciciliot S, Danieli-Betto D, Dobrowolny G, Furlan S, Germinario E, Goto K, Gutsmann M, Kawano F, Nakai N, Ohira T, Ohno Y, Picard A, Salanova M, Schiffl G, Blottner D, Musaro A, Ohira Y, Betto R, Conte D, Schiaffino S (2012) Adaptation of mouse skeletal muscle to long-term microgravity in the MDS mission. PLoS ONE 7:e33232. 10.1371/journal.pone.0033232 22470446 10.1371/journal.pone.0033232 PMC3314659

[14] Kawano F, Nimura K, Ishino S, Nakai N, Nakata K (1985) Ohira Y (2015) Differences in histone modifications between slow- and fast-twitch muscle of adult rats and following overload, denervation, or valproic acid administration. J Appl Physiol 119:1042–1052. 10.1152/japplphysiol.00289.2015 10.1152/japplphysiol.00289.2015 26404615

[15] Arpke RW, Shams AS, Collins BC, Larson AA, Lu N, Lowe DA, Kyba M (2021) Preservation of satellite cell number and regenerative potential with age reveals locomotory muscle bias. Skelet Muscle 11:22. 10.1186/s13395-021-00277-2 34481522 10.1186/s13395-021-00277-2 PMC8418011

[16] Clemens Z, Sivakumar S, Pius A, Sahu A, Shinde S, Mamiya H, Luketich N, Cui J, Dixit P, Hoeck JD, Kreuz S, Franti M, Barchowsky A, Ambrosio F (2021) The biphasic and age-dependent impact of klotho on hallmarks of aging and skeletal muscle function. Elife. 10.7554/eLife.61138 33876724 10.7554/eLife.61138 PMC8118657

[17] Zhang FM, Wu HF, Wang KF, Yu DY, Zhang XZ, Ren Q, Chen WZ, Lin F, Yu Z, Zhuang CL (2024) Transcriptome profiling of fast/glycolytic and slow/oxidative muscle fibers in aging and obesity. Cell Death Dis 15:459. 10.1038/s41419-024-06851-y 38942747 10.1038/s41419-024-06851-y PMC11213941

[18] Markworth JF, Brown LA, Lim E, Castor-Macias JA, Larouche J, Macpherson PCD, Davis C, Aguilar CA, Maddipati KR, Brooks SV (2021) Metabolipidomic profiling reveals an age-related deficiency of skeletal muscle pro-resolving mediators that contributes to maladaptive tissue remodeling. Aging Cell 20:e13393. 10.1111/acel.13393 34075679 10.1111/acel.13393 PMC8208786

[19] Yoshie T, Saito C, Kawano F (2020) Early high-fat feeding improves histone modifications of skeletal muscle at middle-age in mice. Lab Anim Res 36:25. 10.1186/s42826-020-00060-2 32793459 10.1186/s42826-020-00060-2 PMC7414670

[20] Murach KA, White SH, Wen Y, Ho A, Dupont-Versteegden EE, McCarthy JJ, Peterson CA (2017) Differential requirement for satellite cells during overload-induced muscle hypertrophy in growing versus mature mice. Skelet Muscle 7:14. 10.1186/s13395-017-0132-z 28693603 10.1186/s13395-017-0132-z PMC5504676

[21] McCarthy JJ, Mula J, Miyazaki M, Erfani R, Garrison K, Farooqui AB, Srikuea R, Lawson BA, Grimes B, Keller C, Van Zant G, Campbell KS, Esser KA, Dupont-Versteegden EE, Peterson CA (2011) Effective fiber hypertrophy in satellite cell-depleted skeletal muscle. Development 138:3657–3666. 10.1242/dev.068858 21828094 10.1242/dev.068858 PMC3152923

[22] Deepa SS, Van Remmen H, Brooks SV, Faulkner JA, Larkin L, McArdle A, Jackson MJ, Vasilaki A, Richardson A (2019) Accelerated sarcopenia in Cu/Zn superoxide dismutase knockout mice. Free Radic Biol Med 132:19–23. 10.1016/j.freeradbiomed.2018.06.032 30670156 10.1016/j.freeradbiomed.2018.06.032 PMC6405207

[23] Sakuma K, Kinoshita M, Ito Y, Aizawa M, Aoi W, Yamaguchi A (2016) p62/SQSTM1 but not LC3 is accumulated in sarcopenic muscle of mice. J Cachexia Sarcopenia Muscle 7:204–212. 10.1002/jcsm.12045 27493873 10.1002/jcsm.12045 PMC4864138

[24] Pannerec A, Springer M, Migliavacca E, Ireland A, Piasecki M, Karaz S, Jacot G, Metairon S, Danenberg E, Raymond F, Descombes P, McPhee JS, Feige JN (2016) A robust neuromuscular system protects rat and human skeletal muscle from sarcopenia. Aging (Albany NY) 8:712–729. 10.18632/aging.100926 27019136 10.18632/aging.100926 PMC4925824

[25] Szenker E, Ray-Gallet D, Almouzni G (2011) The double face of the histone variant H3.3. Cell Res 21:421–434. 10.1038/cr.2011.14 21263457 10.1038/cr.2011.14 PMC3193428

[26] Saksouk N, Simboeck E, Dejardin J (2015) Constitutive heterochromatin formation and transcription in mammals. Epigenetics Chromatin 8:3. 10.1186/1756-8935-8-3 25788984 10.1186/1756-8935-8-3 PMC4363358

[27] Eason JM, Schwartz GA, Pavlath GK (1985) English AW (2000) Sexually dimorphic expression of myosin heavy chains in the adult mouse masseter. J Appl Physiol 89:251–258. 10.1152/jappl.2000.89.1.251 10.1152/jappl.2000.89.1.251 10904059

[28] Shoji H, Takao K, Hattori S, Miyakawa T (2016) Age-related changes in behavior in C57BL/6J mice from young adulthood to middle age. Mol Brain 9:11. 10.1186/s13041-016-0191-9 26822304 10.1186/s13041-016-0191-9 PMC4730600

