# Supplementary material for: Age-related histone H3.3 accumulation associates with a repressive chromatin in mouse tibialis anterior muscle
Source: J Physiol Sci. 2024 Sep 14;74:41. doi: 10.1186/s12576-024-00935-2 (PMC11401410; doi:10.1186/s12576-024-00935-2)
Supplement: Supplementary file 4 — Additional file 4. Figure S1–S8. Supporting figures and their legends. [file 12576_2024_935_MOESM4_ESM.docx]

Figure S1


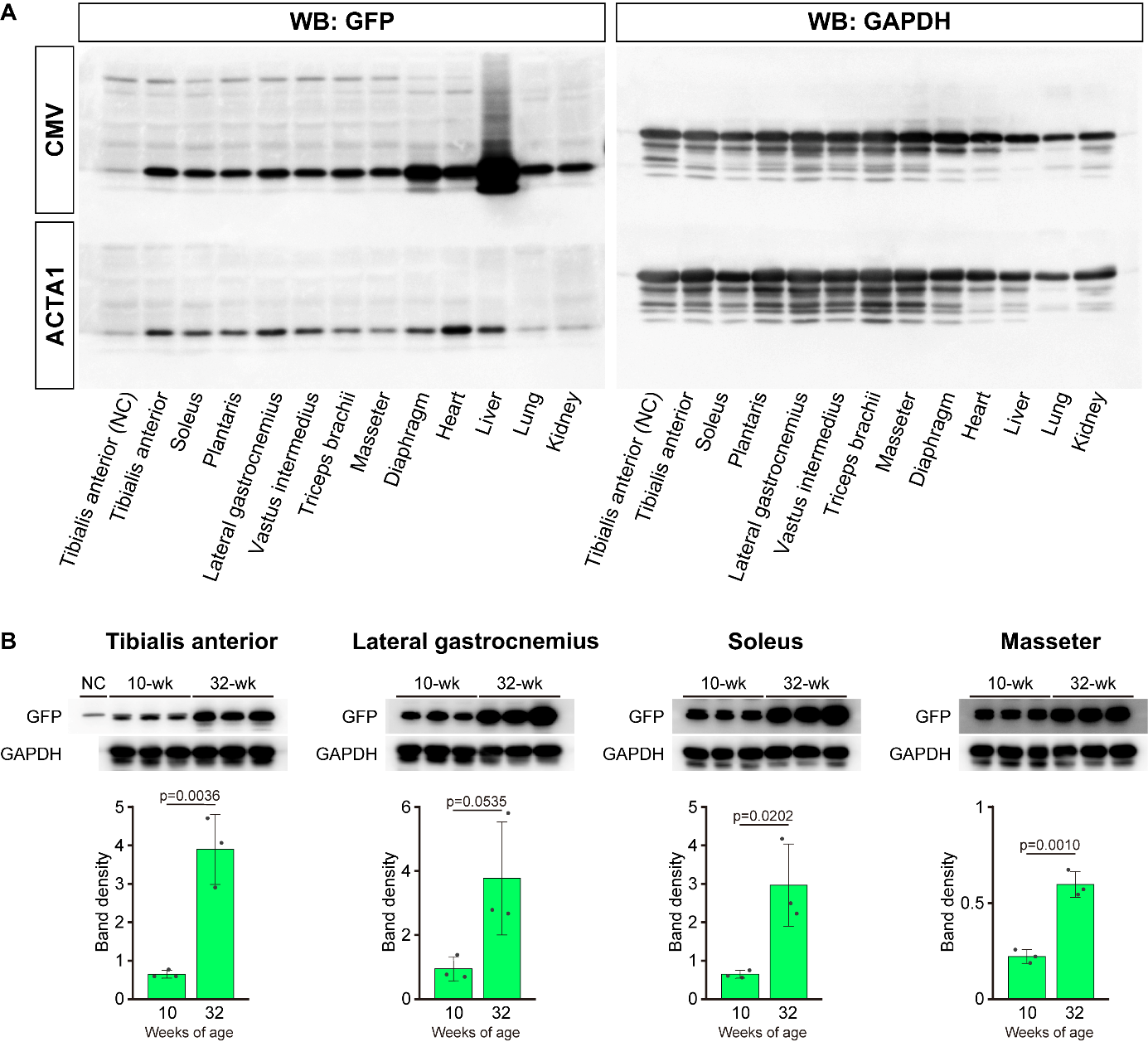


A: Western blotting using GFP and GAPDH antibodies in the mice treated with AAV9 vector expressing EGFP under the control of CMV or ACTA1 promoter. AAV9 vector (2 x 10^11^ vg) was intravenously injected at 8-wk-old. Skeletal muscles and organs were sampled at 10-wk-old. To show the differences of protein levels between CMV and ACTA1 promoters, all samples were blotted on same membrane. B: Comparison of GFP level in tibialis anterior, lateral gastrocnemius, soleus, and masseter muscle between 10- and 32-wk-old (n=3 each). Note that GFP level was significantly enhanced at 32-wk-old compared to 10-wk-old in all skeletal muscles. Mean ± SD.

Figure S2


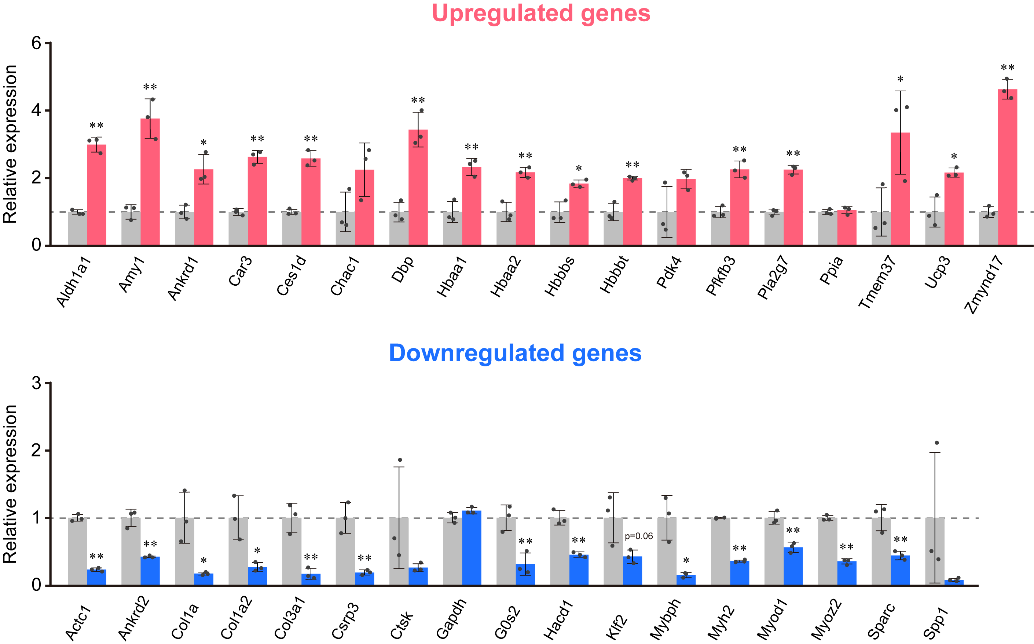


Confirmation of differences in the expression of target genes that were identified by RNA sequencing analysis. The expression of each gene was compared between 8- (grey bars) and 75- (red and blue bars) wk-old. The genes that found the significant difference by Student’s unpaired *t* test were targeted for the further analysis. The data were represented as the relative expression to *Rpl31*. Mean ± SD. * and **: *p* < 0.05 and 0.01 vs. 8-wk-old, respectively.

Figure S3


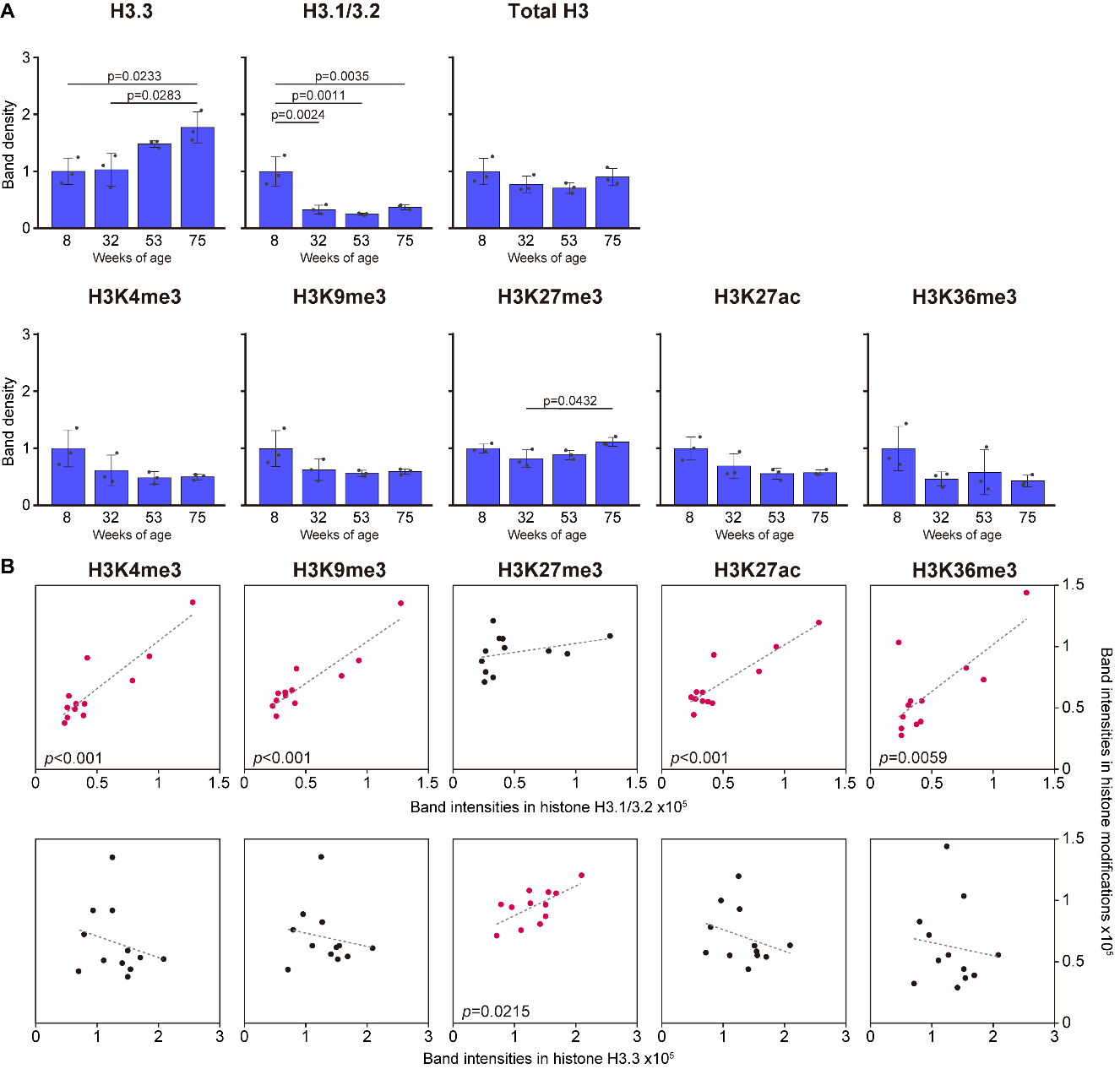


A: Age-related changes in the levels of H3.3, H3.1/3.2, and histone modifications in the tibialis anterior muscle determined by western blotting. Significant differences were examined by one-way ANOVA followed by Scheffe’s *post hoc* test. B: X-Y plots showing the correlation between H3.3 or H3.1/3.2 and histone modifications. Significance of correlation was examined by Pearson correlation.

Figure S4


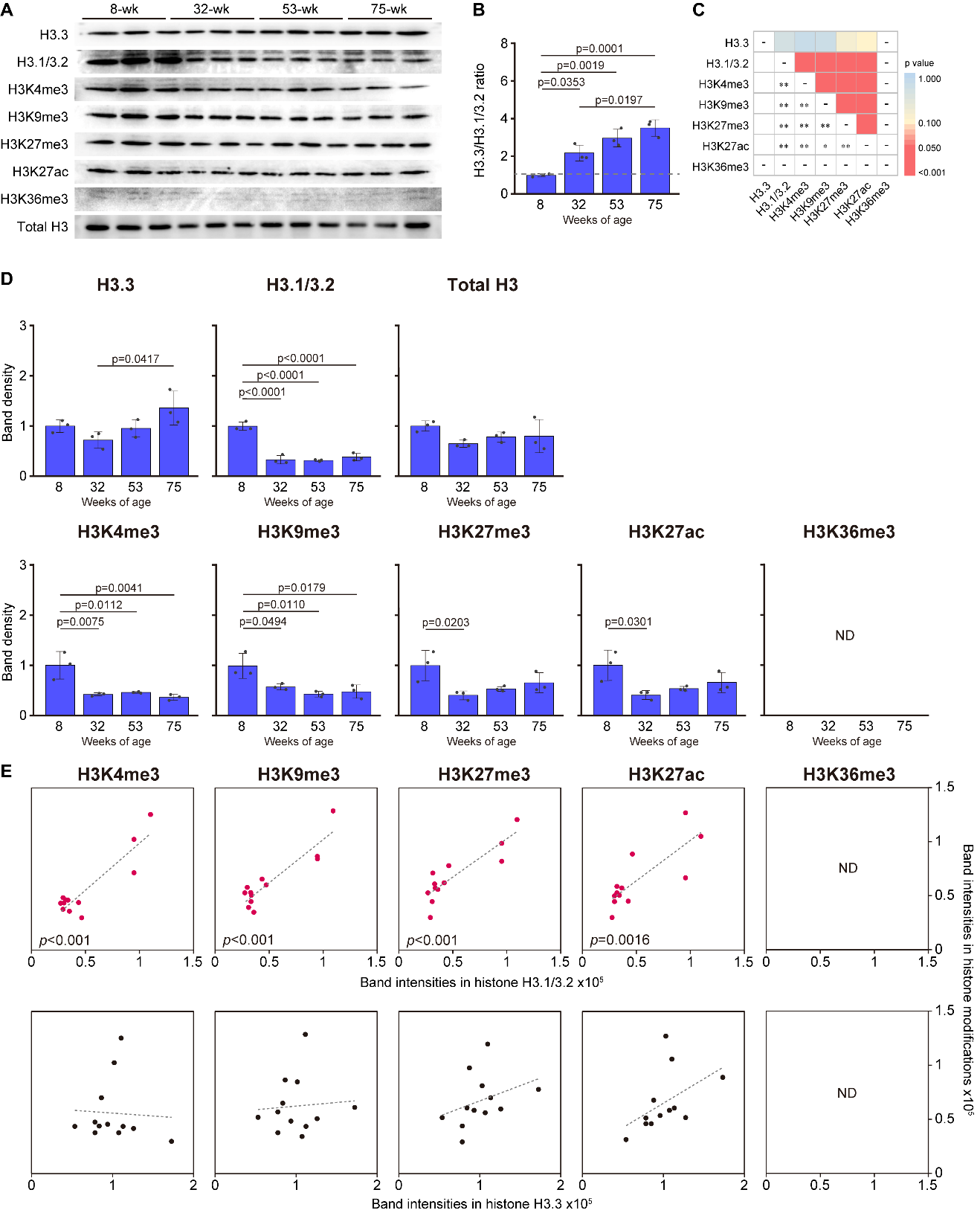


Age-related changes in the levels of H3.3, H3.1/3.2, and histone modifications in the soleus muscle determined by western blotting. A: All blot images. B: H3.3 to H3.1/3.2 ratio at 8, 32, 53, and 75 weeks of age. C: Heat map showing the p values obtained using Pearson correlation between various histone modifications. *: p < 0.05. **: p < 0.01. D: Quantified levels of each histone or histone modifications. Significant differences were examined by one-way ANOVA followed by Scheffe’s *post hoc* test. E: X-Y plots showing the correlation between H3.3 or H3.1/3.2 and histone modifications. Significance of correlation was examined by Pearson correlation.

Figure S5


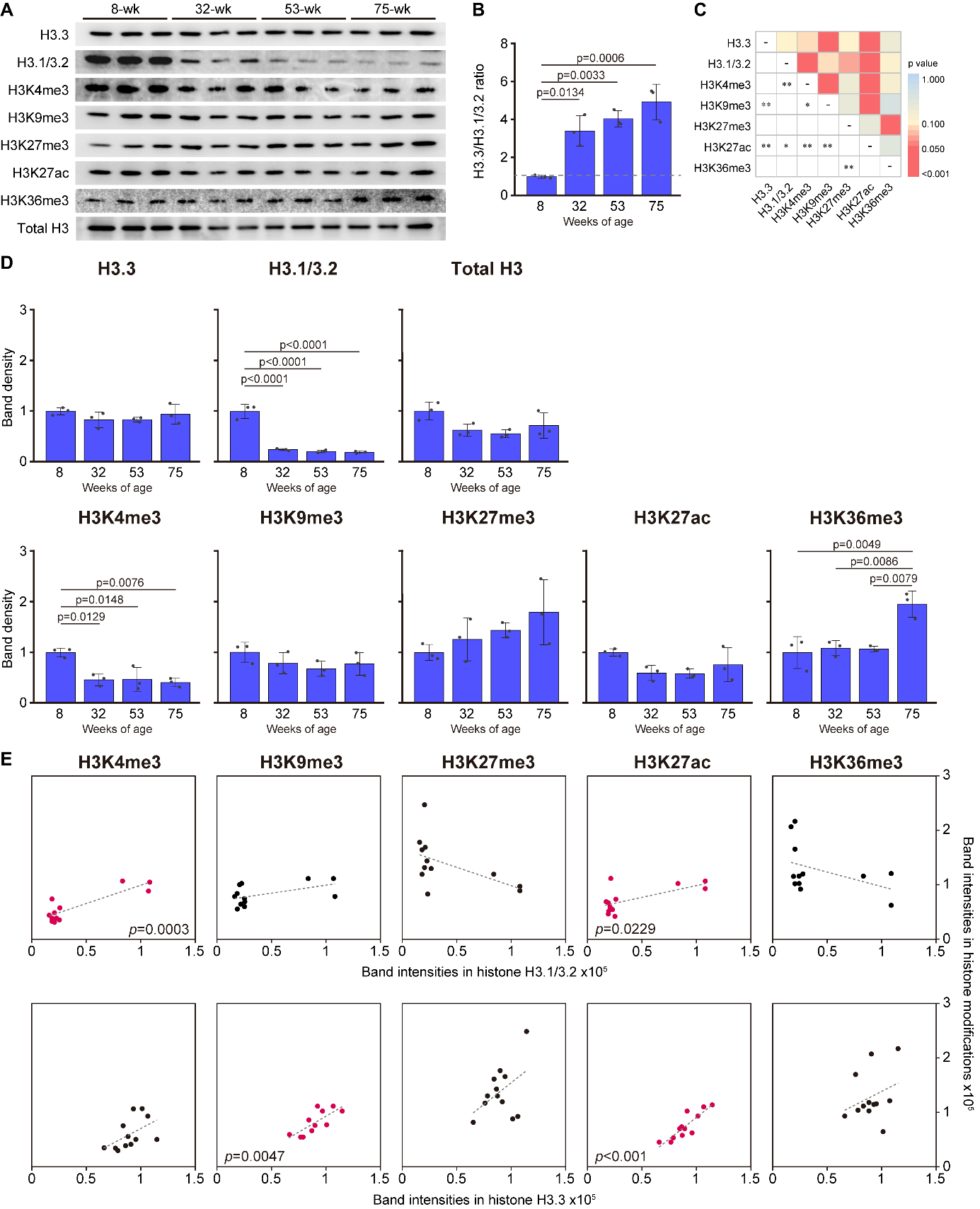


Age-related changes in the levels of H3.3, H3.1/3.2, and histone modifications in the masseter muscle determined by western blotting. A: All blot images. B: H3.3 to H3.1/3.2 ratio at 8, 32, 53, and 75 weeks of age. C: Heat map showing the p values obtained using Pearson correlation between various histone modifications. *: p < 0.05. **: p < 0.01. D: Quantified levels of each histone or histone modifications. Significant differences were examined by one-way ANOVA followed by Scheffe’s *post hoc* test. E: X-Y plots showing the correlation between H3.3 or H3.1/3.2 and histone modifications. Significance of correlation was examined by Pearson correlation.

Figure S6


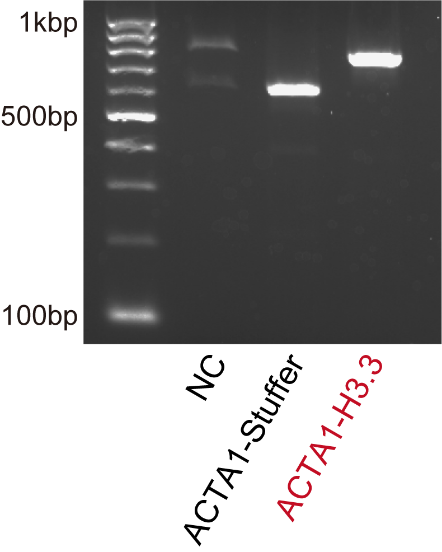


Detection of AAV9 vector persisted in the tibialis anterior muscle at 32-wk-old in Experiment 3. Toal DNA was extracted from the muscle samples combined in each group (n=5 each). PCR analysis was performed with a specific primer set which were designed to span between the ACTA1 promoter and the 3’ polyadenylation signal sequences. A 606 bp product indicates the stuffer sequence, and a 768 bp product indicates the H3f3a sequence. NC, uninjected negative control.

Figure S7


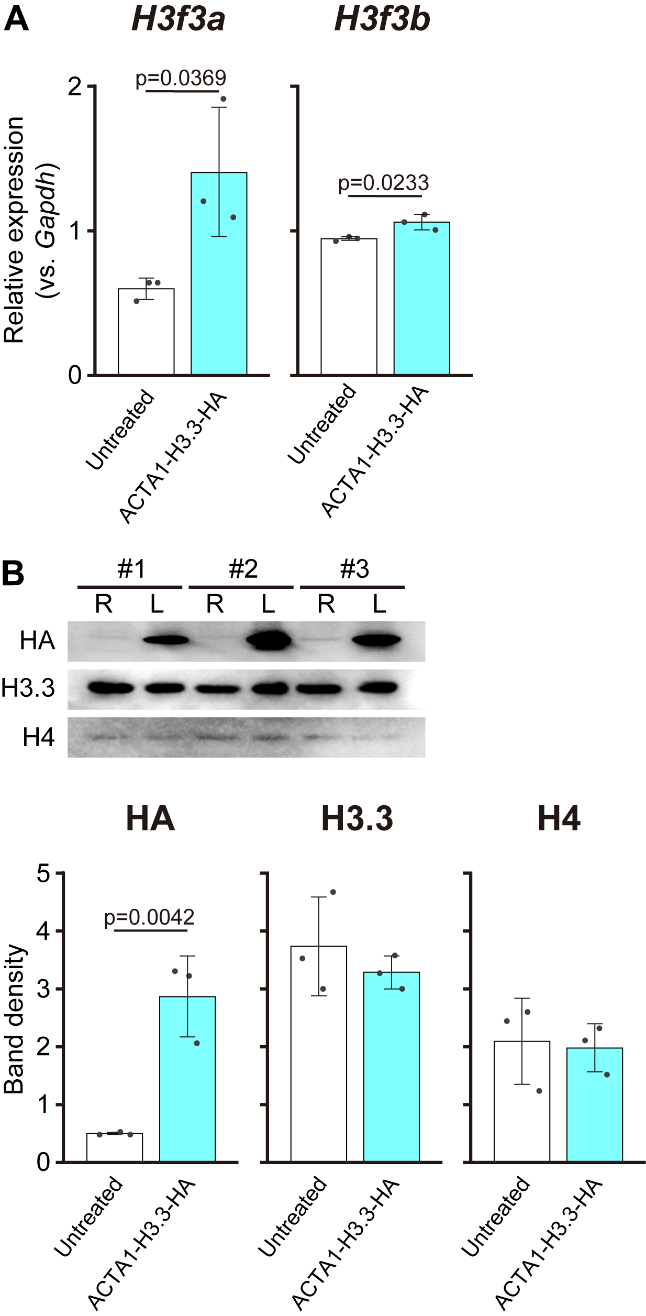


Effects of AAV9 vector carrying ACTA1-H3.3-HA intramuscular administration on gene expression and protein production. *H3f3a* and *H3f3b* mRNA expression (A) and protein levels of H3.3 analyzed by western blotting (B) in the untreated and vector-injected tibialis anterior muscles. The vector was designed to express *H3f3a* mRNA with an HA tag-coding sequence at the 3’ end. The AAV9 vector (1 x 10^11^ vg) was injected into the left tibialis anterior muscle of 8-wk-old mice. The contralateral side (right) remained untreated as the control. Muscle sampling was performed 4 weeks after the injection.

Figure S8


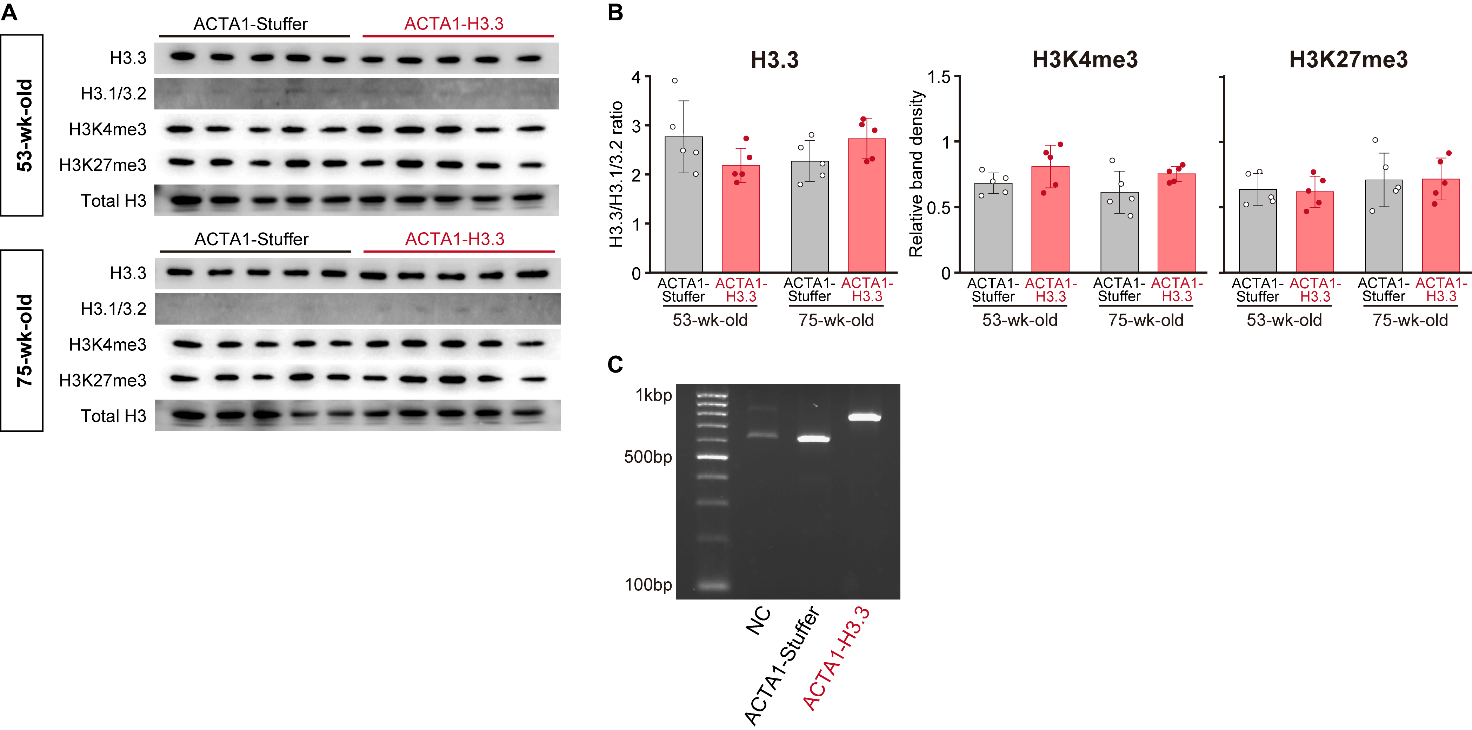


The results in the western blotting analysis for H3.3, H3.1/3.2, and histone modifications in the tibialis anterior muscles of mice treated with AAV9 vector carrying ACTA1-Stuffer or ACTA1-H3.3 sequences. AAV9 vector (8 x 10^11^ vg) was intravenously injected at 23-wk-old. Skeletal muscles and organs were sampled at 53- and 75-wk-old. C: Detection of AAV9 vector persisted in the tibialis anterior muscle at 75-wk-old. See Fig. S6 for details of the primer design.
